# “I took it off most of the time 'cause I felt comfortable”: unmasking, trusted others, and lessons learned from a coronavirus disease 2019 reinfection: a case report

**DOI:** 10.1186/s13256-021-03033-8

**Published:** 2021-11-11

**Authors:** Jacinda K. Dariotis, Stephanie M. Sloane, Rebecca Lee Smith

**Affiliations:** 1 Department of Human Development and Family Studies, The Family Resiliency Center, College of Agricultural, Consumer and Environmental Sciences, Beckman Institute for Advanced Science and Technology, The University of Illinois at Urbana-Champaign, 904 W. Nevada Street, 904 W. Nevada Street, Urbana, IL 61801 USA; 2 Department of Population, Family, and Reproductive Health, The Johns Hopkins University Bloomberg School of Public Health, Baltimore, MD USA; 3 Department of Pathobiology, College of Veterinary Medicine, Carl R. Woese Institute for Genomic Biology, Carle-Illinois College of Medicine, The University of Illinois at Urbana-Champaign, VM BSB 2418, Urbana, IL 61801 USA

**Keywords:** COVID-19, Risk-taking, Decision-making, Stigma, Social pressure, Case report

## Abstract

**Background:**

Severe acute respiratory syndrome coronavirus 2 reinfection prevalence is unknown. It is essential to understand reinfection symptoms and, importantly, the lived experience.

**Case presentation:**

Case study design is the best method for understanding this contemporary pandemic and rare occurrence of reinfections. A 19-year-old White Non-Hispanic woman presented with presumed severe acute respiratory syndrome coronavirus 2 reinfection 6 weeks after initially mild symptomatic infection and consistent repeat negative results. Real-time reverse-transcription polymerase chain reaction from saliva was used for detection. Twice-weekly saliva samples were collected (a) before initial infection, (b) resumed on day 10 after initial infection until reinfection was detected, and (c) resumed on day 10 post-reinfection. A 1.5-hour virtual interview was conducted, transcribed, and independently analyzed by two researchers. Four themes emerged: (1) perceived invincibility or inevitability and subsequent immunity increases risk of transmission via inconsistent preventive behaviors; (2) normalcy desires, trusted others, and implicit social pressures to not wear masks and distance increase one’s coronavirus disease 2019 risk; (3) physical symptoms are more severe with reinfection compared with first infection; and (4) mental health sequelae (trauma and stigma) are more severe and enduring than physical health outcomes.

**Conclusions:**

Unmasked social interactions contradicting public health recommendations were rationalized by social circle members with heavy reliance on feeling asymptomatic, lacking a positive test (testing negative or not testing), or attributing symptoms to allergies. Stigma of testing positive and consequences of not conforming to social group behaviors is overwhelming and creates pressure to take risks. This case study provides insights and lessons learned relevant for public health messaging and continued preventive behaviors.

## Background

As of 22 February 2021, over 112 million people globally [1] and nearly 28 million in the USA [2] have tested positive at least once for severe acute respiratory syndrome coronavirus 2 (SARS-CoV-2). Reinfection prevalence is unknown. As of 20 December 2020, a total of four viral genome sequence confirmed reinfection cases have been reported in the empirical literature (Hong Kong, Nevada, Belgium, Ecuador) [3]. Of these four, two presented worse symptoms upon reinfection [4]. Cases occurred within less than 90 days (one within 48 days) of initial infection [3]. With emergency vaccine dissemination, anticipated decreases in masking and distancing, and variants of concern that may reinfect persons who already had coronavirus disease 2019 (COVID-19), reinfection has become a larger concern than initially thought [4].

Psychosocial factors have been ignored in previous reinfection studies that exclusively present biological factors. It is essential to understand reinfection not only in terms of symptoms but, more importantly, the lived experience. The goal of this case study is to describe and explain the lived experience of a participant with COVID-19 reinfection. Given that COVID-19 reinfection cases are rare, their study is time-sensitive given the just-in-time nature of this pandemic, and the descriptive and exploratory focus of the case study methodological approach is best suited to understand this phenomenon [5–7]. Further, this case study provides insights and lessons learned relevant for public health messaging and continued preventive behaviors.

## Case presentation

### Case history

**Fig. 1:**
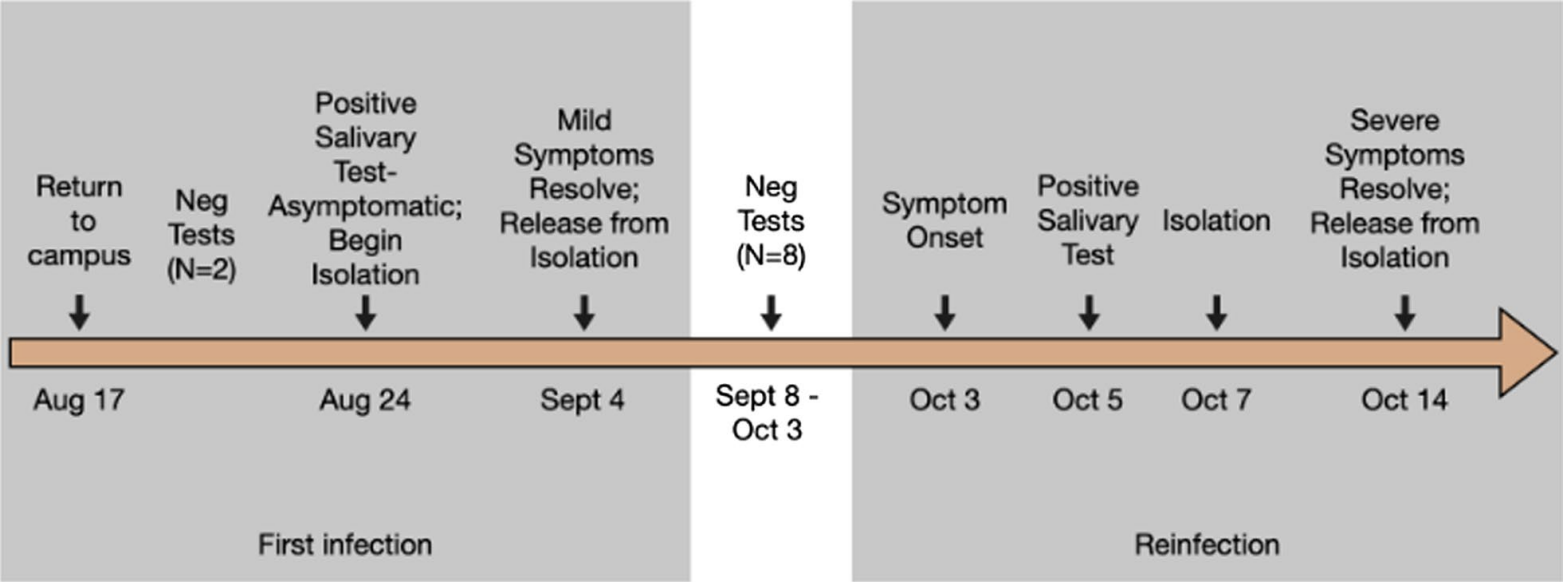
Timeline of testing, symptom onset, isolation, and retesting

A 19-year-old White Non-Hispanic female student with no underlying health conditions and no past psychiatric illness attending a Midwestern public university moved to campus on 17 August 2020 to begin the 2020–21 academic year. All students were required to take two saliva virus (PCR) tests weekly. The timeline of testing, symptom onset, isolation, and retesting is shown in Fig. 1. She had two negative results during her first 7 days on campus and first tested positive on 24 August with no symptoms. She isolated in an apartment with three female friends who also tested positive (creating an isolation “Pod”). During isolation (25 August–3 September), she reported mild physical symptoms consistent with viral infection (headache, aches and pains, fatigue, shortness of breath, chest pain, and diarrhea). She reported that her Pod peers experienced worse physical symptoms than herself. Her symptoms resolved by the end of isolation, and she resumed testing. She consistently tested negative twice weekly from 8 September until 5 October, when she received her second positive result. Two days prior to her second positive test, she began exhibiting many of the same symptoms she experienced with her first infection. Compared with her previously mild symptoms, her reinfection symptoms were moderate to severe and included more impacts (severe: coughing and loss of appetite; moderate: stuffy nose, fatigue, diarrhea, abdominal pain, and loss of sleep; mild: shortness of breath and difficulty thinking). She began self-isolation on 6 October (e.g., stopped sharing a bathroom with roommates) and isolated in a hotel 7–14 October. Her physical symptoms resolved during isolation except for a persistent cough.

### Procedures

The participant provided written consent to be part of this case study and for the findings to be published. The UIUC Institutional Review Board confirmed the case study status of this work.

Saliva for each test was collected via an observed self-collection, in which the participant would drool into a 50 mL conical tube. A direct saliva RT-qPCR process was used for detection [8]. All positive samples were retested, and results were reviewed by laboratory personnel. Any positive results following isolation were also reviewed by the ordering physician to confirm reinfection as opposed to recurrent noninfectious positive results.

The interview was conducted via video conferencing by a trained mixed methodologist. A semi-structured interview [9] protocol was used to ensure major topics were discussed including: timing of testing and positive results; degree of symptom severity (scale: none, slight/mild, moderate, severe, very severe) at first infection and reinfection; information sources; self, peer, and family members’ perceptions, attitudes, and behaviors; mental health concerns; and advice for peers, family members, older adults, health officials, and others. The 90-minute interview was audio recorded and transcribed.

The case study—an inductive qualitative analytic approach—is justified given the exploratory nature of this contemporary and real-time wicked problem [6, 7]. The goal of this case study is to describe and explain the lived experience of COVID-19 reinfection. A holistic analysis was conducted. First, the case context is described regarding chronological events. Then, themes emerging from the case are detailed. Last, the themes are interpreted, and parallels are drawn to other public health epidemics. Two researchers independently read the transcript and initially identified major and subthemes. Through an iterative process of reviewing summaries and discussions, the final version of themes and descriptions were agreed upon [10, 11].

## Results

Four themes emerged from this case study.

### Theme 1: Perceived invincibility or inevitability and subsequent immunity increases risk of transmission via inconsistent preventive behaviors

Perception played an important role in this participant’s COVID-19-related preventive behaviors. When the pandemic first impacted her life, the severity became reality when she was not allowed to return to campus after spring break (March 2020). Rather, she was forced to live with her family as a result of the stay-at-home order March through June 2020. During the summer, her social interactions were relatively controlled: outside gatherings with distancing and limited to friends who claimed to heed public health recommendations of wearing masks and limiting social interactions.

Despite the excitement of returning to school, she arrived at campus believing becoming infected with the virus was inevitable. As she stated: “*I was really excited [to return to school]. Like obviously very nervous too 'cause I knew the chances of me getting it were so high. I knew I was bound to get it at least once.”*

She was eager to leave her parents’ home and return to school despite the increased risk of contraction. She understood the seriousness of the virus and followed many of the guidelines for prevention (e.g., mask-wearing to varying degrees, keeping her social circle small). She, however, felt strongly about hanging out with friends and not letting fear determine all of her behaviors. As she described,

*I can’t not live my life because I’m in fear all the time. I have to learn to live with this virus eventually. Like obviously just be very cautious. Don’t do anything that could put you at risk. But, you can’t just sit in your room alone all night and not go out and see anyone 'cause then what’s even the point of being here?*

Perceived illness inevitability and mindset that one cannot live in fear promoted inconsistent preventive behaviors, which was further compounded by an *initial* disbelief that reinfection was possible and *subsequent* belief that immunity would last at least 90 days. As she stated “*No, I wasn’t concerned about getting reinfected. I didn’t think it was possible, especially within the 90 days*.” After her first isolation, she engaged in riskier behaviors compared with when she first returned to campus. She was less disciplined about wearing her mask around friends, even in larger groups.

This placed her at greater risk because she interacted with friends who were convinced they were physically robust and could not get sick. These friends were not consistently testing or engaging in preventive behaviors because they were asymptomatic and felt invincible. When she was reinfected, her friend tested and was positive. As she remembered,

*Looking back at it now, they [friends] were having symptoms before I was. So I think I got it from them, not them getting it from me. They went home and …when they came back, they were coughing. And they kept telling me ‘it's just a cough. It’s allergies.’ His cough wasn’t as bad as mine was. It wasn’t as frequent and as loud. So he was like, ‘really, I can’t get [sick].’ He had been exposed a lot of times and had never gotten it. So this was his first time having it even though he had known so many people at the beginning of the year who had it. So he was like ‘I can’t get it.’*

These varied perceptions—inevitable illness, invincibility, and immunity—coupled with inconsistent risk-taking behaviors creates a perfect storm for transmission, infection, and reinfection. This participant, after reinfection, developed an appreciation for testing, noting *“I think the testing is really great and it makes me feel like maybe even though I have had it makes you feel a little bit more safe.”*

### Theme 2: The perils of normalcy desires, trusted others, and implicit social pressures to not wear masks and physically distance

The participant expressed a clear desire to protect her family (and herself, especially after her second infection) from infection. However, her desire for close proximity to her friends, without masks, was a stronger determinant of her behavior. She admits that she and her peers were so focused on establishing a sense of normalcy that they risked becoming sick. As she aptly described:

*We were just trying to live normal life, like last year. We wanted so badly to have the same experience that we did last year and for it not to be different. We tried to do everything we could to make it like that. I guess in the process we weren’t being the smartest, obviously since we got it.*

The participant spoke about feeling safe around her trusted friends. She tried to limit the size of her social circle. She spent most of her time with female housemates and with male friends who lived in one house. She did not visit other friends’ apartments, houses, or dorms. She assumed each person in her trusted circle was taking the same precautions she was. As she described:

*I was wearing a mask in common areas but in their personal rooms where we were hanging out I took it off most of the time 'cause I felt comfortable. I didn’t feel like they were exposed. I didn’t think I was exposed. But they were also not really wearing masks. I didn’t really do social distancing that much with them either.*

The level of preventive behaviors in the two houses shifted over time. She described first following the public health recommendations in areas with more traffic and not in personal rooms:

*I guess at times I just feel safe. I’m gonna take it [mask] off to talk to people and I’ll put it on when I go walk and there’s no one in the halls. Sometimes in the personal rooms it would be really crowded, and I would hold my mask. I was wearing it but then I’d pull it down and talk to people*.

Behaviors progressively shifted from strict mask-wearing and distancing to greater exceptions being made, especially at the time of her reinfection. She admitted rarely wearing masks or practicing distancing with her trusted circle. They had an understanding that, if everyone was comfortable, they would all remove their masks.

*It [wearing masks] started out really strict. It’s gotten progressively lenient and it’s more of a comfort base now. If everyone in the room that you’re in is comfortable without masks on, then you take them off. But if someone’s not comfortable, you keep them on, sure. But at the beginning, it was everyone wears masks at all times unless you’re in your room or you’re in a friend’s room who says you can take it off.*

She trusted her close circle of friends to adhere to prevention guidelines *outside* the circle in order to keep themselves safe, which in turn would keep her safe. Because her network of friends perceived they were not interacting with others outside their circle and therefore assumed minimal risk of infection, they stopped regular testing. As she described:

*Because we don’t go to that many buildings, I don’t think that they get tested that often, unfortunately. So I just think they don’t really care as much. They do [get tested] but not as often as I do. [It’s] not like they don’t do it. They just go when they wanna go. They don’t go when they’re required to go… I didn’t really know they weren’t complying until I realized that I probably got it from one of them. For the second time, I just think they don’t really care, they don’t really want to take the time out of their day to go. Think it’s kind of inconvenient sometimes.*

She and her friends rarely wore masks when together because they felt “comfortable” that none of them had been exposed elsewhere. When she reflected back on September and October, she admitted that she did not know how safe her male friends were being and concluded that she most likely contracted the virus from them both times. Inconsistent testing coupled with pressures to relax preventive behaviors increases the risk of transmission.

### Theme 3: Physical symptoms are more severe with reinfection compared with first infection

This participant reported experiencing no symptoms before her first positive test. Testing regardless of symptomatology likely increased virus detection among presymptomatic individuals. She also offered that, despite having a headache and mild aches after her first positive test, she would not have known she had COVID-19 without the positive test result.

She was asked to rate the severity of a list of symptoms during her first infection and reinfection using a scale of “not at all” to “very severe.” As shown in Table 1, she did not experience most symptoms during first infection and reported only mild shortness of breath/ trouble breathing, chest pain, and diarrhea and moderate fatigue during isolation.

**Table 1:** Physical symptom severity ratings at first and reinfection

Physical symptom	First infection	Reinfection
High temperature/fever	Not at all	Not at all
A new continuous cough	Not at all	Severe
Sore throat	Not at all	Not at all
Shortness of breath or trouble breathing	Mild	Mild
Chest pain	Mild	Not at all
Fatigue	Moderate	Moderate
Loss of sense of smell or taste	Not at all	Not at all
Diarrhea	Mild	Moderate
Abdominal pain	Not at all	Moderate
Loss of appetite	Not at all	Severe
Difficulty thinking	Not at all	Mild
Loss of sleep	Not at all	Moderate

She remembered that her symptoms began on 3 October, 2 days before her second positive test. She reported having a severe cough, a stuffy and runny nose, mild shortness of breath, moderate fatigue, moderate diarrhea, moderate abdominal pain, severe loss of appetite, mild difficulty thinking, and moderate loss of sleep. At the time, she did not attribute her symptoms to a COVID-19 reinfection because she believed she could not become reinfected for at least 90 days.

*So on the 3rd and the 4th [of October] I was coughing a lot. I had a lot of symptoms that I didn’t realize were symptoms: coughing, stuffy nose, runny nose. And just not feeling 100%. I thought that they were allergies or that they were just the weather changing 'cause last year I had also gotten a cold and I had a cough for so long… I had an appointment on the 5th with a doctor from home, a Skype call, and they told me they thought they were just allergies too. Not Corona.*

Her saliva test on 5 October revealed she was positive for the second time. The symptoms mimicking common cold and allergy symptoms meant that this participant and her primary care physician did not attribute her symptoms to COVID-19 without positive test results.

### Theme 4: Mental health sequelae are more severe than physical health outcomes—trauma, stigma, and creative coping with isolation pods

Humans are social and emotional beings. Social isolation is a long-standing form of torture. The Public Health Department mandated 10-day physical isolation requirement post-positive test result was the hardest part of this woman’s entire COVID-19 experience. During her first isolation, she shared a short-term apartment rental with three COVID-positive friends. This creative coping strategy protected her from the full magnitude of social and physical separation from peers and family. She reported being anxious and stressed during her initial isolation, but extremely anxious and traumatized as a result of reinfection. Given her limited resources during reinfection, for a week she had to isolate alone in a hotel room, funded by the local Public Health Department. As she described:

*I cried a lot that whole week. I was really anxious, and I’ve never been an anxious person. I started to become extremely anxious all the time. I was always sad. I was a lot more anxious and sad than I was the first time and than I ever was even in my life.*

Even though she became aware of another woman isolating at the same time in the hotel and arranged to watch a movie in the evenings, the cumulative impact of two isolations and distancing recommendations is long term and traumatizing.

*The mental impact has obviously been a lot worse ‘cause it’s lasted a lot longer than the physical symptoms. I had them [physical symptoms] for a few days and now they’re not as bad. [But] the mental stays with me throughout and it’s probably never gonna go.*

She characterized the traumatization as follows: “*I would never wish this on anyone else. What happened to me was awful and I would never wish on my worst enemy to get it twice; to even get it once.*” The continuous and compounding social stigma she experiences exacerbates the mental health consequences of COVID-19. As she noted “*I do think I’ve been a little stigmatized. About what happened to me.*” Testing positive negatively impacted her friendships. At the time of the interview, 1 month after testing positive the second time, she was still being stigmatized. Nearly 2 weeks following her second isolation, she continued to feel anxiety about her friendships and in social situations.

*“I get really anxious and nervous not just about COVID but I feel like it’s affected my friendships. I’ve been getting really anxious about friendships like when people are upset with me or mad at me. I think that it’s affected everything in my life… I’ve been a lot more cautious, obviously about wearing my mask. I wear it everywhere. I barely ever take it off and when I do, after the fact if something happens, I get really anxious about it.*

## Discussion

This case presents a rare opportunity to deepen our understanding of a person’s experience with COVID-19 upon initial infection and reinfection. In another context, this participant would not have undergone testing and therefore would not have known of her initial positive status. This is consistent with warnings that reinfection numbers are grossly underestimated.

Four main themes emerged: (1) perceived invincibility or inevitability and subsequent immunity increases risk of transmission via inconsistent preventive behaviors; (2) a desire for normalcy, assumed health and safety of trusted others, and implicit social pressures discourage mask wearing and distancing; (3) physical symptoms are more severe with reinfection compared with first infection; and (4) mental health sequelae (trauma and stigma) are more severe and longer lasting than physical health outcomes.

The COVID-19 pandemic poses a tension between preventive behaviors (specifically mask-wearing and distancing) and risk perceptions (e.g., inevitability or invincibility) and the importance of face-to-face social interaction. This is particularly challenging for people who seek, are accustomed to, or expect an active social life. Observing trusted friends and family members not wearing masks or distancing, coupled with a desire to return to normalcy, creates expectations and pressures to relax preventive behaviors.

Testing is psychologically complex. For some people, it may provide false assurance that one is currently negative and justifies unprotected socializing. Regular testing is a reassurance for people who perceive it as keeping them and others safe. For others, negative status is assumed if they present no symptoms and avoiding testing means they do not risk an asymptomatic positive that will restrict their behaviors.

Numerous paradoxes were revealed in this case study that parallel other epidemics. The unintended consequences associated with sources of false security heightens the significance for learning about the lived experience of reinfection. This case study provides insights and lessons learned relevant for public health messaging and continued preventive behaviors.

First, symptoms were rated worse during reinfection relative to initial infection. This virus violates the assumption that immunity from an initial infection would result in a less severe second illness. Given this increase in severity, combined with the low likelihood of detecting reinfections in the USA, the possibility of reinfection should not be underplayed or deemphasized as a rare occurrence. This is particularly important as vaccines are distributed, as those who are vaccinated may hold similar beliefs about protection as those who have previously been infected. Mask-wearing and distancing should be underscored as important even for recovered individuals.

Second, rationalizations were made as a coping strategy to justify unprotected, close social interactions. These included feeling asymptomatic and testing negative or not testing at all, which provided a false comfort, as did ignoring symptoms or explaining them as allergies or a cold.

Third, the stigma associated with testing positive and social ramifications of not gathering or not succumbing to pressures to go without masks and distancing have deleterious effects on people. The long-lasting mental health consequences of testing positive or being isolated from others motivated creative coping and, at times, elevated risk-taking behaviors. Isolation pods and pods of trusted friends helped maintain social connections. However, trusting others to practice preventive behaviors to keep oneself and others safe was an ineffective strategy for wellness. Lack of trust of the virus should supersede discomfort of insisting friends and family wear masks and maintain their distance.

The loss of social interaction and other perceived losses potentially outweighs the loss of life as behavioral motivators. The unintended consequences of the synergistic effects of wanting normalcy, trusting others, and innate desire for social connection may be (a) additional waves of infection spikes; (b) massive reductions in preventive behaviors immediately upon vaccination because of a lack of understanding about reinfection; and (c) continual spikes or high levels of infection and reinfection during and after vaccine distribution. Although this is a case study of one participant, her insights warrant consideration and are based on interactions with grandparents, parents, and peers. More mixed methods research is needed to understand the prevalence of reinfection and differential motivations for preventive behaviors to begin to solve this wicked problem.

## Conclusions

Unmasked social interactions contradicting public health recommendations were rationalized by social circle members with heavy reliance on feeling asymptomatic, lacking a positive test (testing negative or not testing), or attributing symptoms to allergies. Stigma of testing positive and consequences of not conforming to social group behaviors is overwhelming and creates pressure to take risks. This case study provides insights and lessons learned relevant for public health messaging and continued preventive behaviors for current and future epidemics and pandemics.

## Data Availability

To maintain confidentiality and privacy of the case study participant, data will remain protected. Additional coding information, however, can be made available from the corresponding author upon reasonable request.

## Abbreviations

**SARS-CoV-2**: Severe acute respiratory syndrome coronavirus 2
**RT-qPCR**: Real-time reverse-transcription polymerase chain reaction
PCR: Polymerase chain reaction
UIUC: University of Illinois at Urbana-Champaign
